# Identification of MST1 as a potential early detection biomarker for colorectal cancer through a proteomic approach

**DOI:** 10.1038/s41598-017-14539-x

**Published:** 2017-10-27

**Authors:** Jiekai Yu, Xiaohui Zhai, Xiaofen Li, Chenhan Zhong, Cheng Guo, Fuquan Yang, Ying Yuan, Shu Zheng

**Affiliations:** 1 Cancer Institute (Key Laboratory of Cancer Prevention and Intervention, China National Ministry of Education), The Second Affiliated Hospital, School of Medicine, Zhejiang University, Hangzhou, 310009 China; 2 Department of Medical Oncology, The Sixth Affiliated Hospital of Sun-Yat Sen University, Guangzhou, 510655 China; 3 Department of Medical Oncology, The Second Affiliated Hospital, School of Medicine, Zhejiang University, Hangzhou, 310009 China; 4 Proteomic Platform, Institute of Biophysics, Chinese Academy of Sciences, Beijing, 100101 China; 5 Research Center for Air Pollution and Health, School of Medicine, Zhejiang University, Hangzhou, 310058 China

## Abstract

Colorectal cancer (CRC) is a common malignant neoplasm worldwide. It is important to identify new biomarkers for the early detection of CRC. In this study, magnetic beads and the Matrix-assisted laser desorption/ionization time-of-flight mass spectrometry (MALDI-TOF-MS) platform were used to analyse CRC and healthy control (HC) serum samples. The CRC diagnosis pattern was established to have a specificity of 94.7% and sensitivity of 92.3% in a blind test. The candidate biomarker serine/threonine kinase 4 (STK4, also known as MST1) was identified by Tandem mass spectrometry (MS/MS) and verified with western blotting and enzyme-linked immunosorbent assay (ELISA). The results indicated that there was a higher concentration of MST1 in HC subjects than stage I CRC patients for the early detection of CRC and a lower concentration in stage IV patients than in other CRC patients. The sensitivity and specificity of MST1 combined with carcinoembryonic antigen (CEA) and faecal occult blood test (FOBT) in diagnosis of colorectal cancer were 92.3% and 100%, respectively. Additionally, low MST1 expression was associated with the poor prognosis. These results illustrate that MST1 is a potential biomarker for early detection, prognosis and prediction of distant metastasis of CRC.

## Introduction

Colorectal cancer (CRC) is the third most common cancer, and it is associated with the second highest malignancy mortality worldwide. Each year, approximately 120,000 CRC cases are newly identified, and approximately 50,000 CRC patients die. The incidence of CRC is increasing each year, and the age of CRC patients is decreasing^1,2^. Therefore, CRC has become a major public health issue. Although there has been great progress in diagnosis and therapy, the therapeutic effectiveness requires improvement, and the five-year survival rate is only approximately fifty percent^3^. The prognosis of CRC at an early stage is much better, and its five-year survival rate can reach ninety to one hundred percent^4,5^. Effective detection of early CRC patients is difficult because they rarely experience uncomfortable symptoms. If the CRC could be detected in early stages, the overall treatment effectiveness and the overall survival rate would be improved^6^. The faecal occult blood test (FOBT)^7^ and serum diagnostic biomarkers such as carcinoembryonic antigen (CEA)^8^ and carbohydrate antigen 19-9 (CA19-9)^9^ have been used for CRC diagnosis, but the accuracy of the FOBT and these biomarkers requires improvement. Alternatively, colonoscopy can significantly improve the diagnosis of CRC. However, this diagnostic method has its shortcomings, such as cost, risk, and inconvenience^10^. In all, it is vital to discovery new CRC biomarkers with high specificity and sensitivity.

Proteomics technology has been used for the detection and validation of protein biomarkers in many kinds of cancer diseases, such as gastric cancer, ovarian cancer, lung cancer, pancreatic cancer, colorectal cancer and breast cancer^11–16^. Matrix-assisted laser desorption/ionization time-of-flight mass spectrometry (MALDI-TOF-MS) is a high-throughput technique that can analyse complex biological specimens and detect multiple protein changes simultaneously with high sensitivity and specificity^17–20^. Combination of magnetic beads and MALDI-TOF-MS is a new method and platform that can be used to identify new cancer biomarkers and other types of disease^21–25^. Therefore, this method is suitable for detection of serum protein biomarkers of CRC.

MALDI-TOF-MS, like Surface-enhanced laser desorption/ionization (SELDI-TOF-MS), can provide only the molecular weight of biomarkers, thus restricting the exploration for clinical biomarkers^26^. Therefore, separation, purification and verification of the candidate biomarkers are also necessary for clinical application. Some protein biomarkers found by SELDI-TOF-MS have been identified by many different methods. In the separation process, other high-abundance proteins usually mask target proteins with low abundance. The rapid acetonitrile (ACN)-based extraction method can be applied to remove high-abundant proteins and enrich low-abundant target proteins from serum^27^. Separation and purification of a protein from complex biological specimens is a complicated procedure. Solid phase extraction (SPE), high-performance liquid chromatography (HPLC) and tricine-SDS-PAGE are the main technologies used to separate and purify low-abundance proteins from serum, urine or tissue lysate^28,29^. After the identification of the biomarker, verification is used to verify the different expression of the proteins through immune methods or other quantitative approaches. Owing to the difficulty of designing an antibody for a peptide, the western blotting method is usually adopted to verify the protein containing the peptide.

In our previous research, we have adopted MALDI-TOF-MS for the screening of cancer biomarkers, and some biomarkers of oesophageal cancer^30^, papillary thyroid cancer^31^, and colorectal cancer^32^ have been successfully discovered. In this paper, we carried out further research on the identification of CRC biomarkers and performed validation with western blotting and enzyme-linked immunosorbent assay (ELISA).

## Results

### Biomarkers and the diagnostic pattern for CRC and healthy control

After filtering the noise and clustering, one hundred and seventy-three peak clusters from 1 kDa to 100 kDa were ranked by *p* values from nonparametric tests. After Wilcoxon rank-sum tests tested the relative signal strength, the Support Vector Machine (SVM) approach was used to identify the combination pattern with the maximum accuracy. Sixty-three healthy control (HC) and 89 CRC samples were selected to form the training test group. The remaining 65 serum samples, as a blind test set, were analysed on the second day to test the SVM pattern.

The top-scored 10 peaks (*p* < 10^−5^) were selected, and the area under the curve (AUC) of these markers were calculated (AUC > 0.78). Then, changes in the 10 markers were evaluated in CRC and HC samples. Markers 1781 Da, 1868 Da and 1694 Da were expressed at higher levels in the CRC than in the HC samples. Otherwise, markers 2084 Da, 1947 Da, 6856 Da, 1951 Da, 2886 Da, 2073 Da and 4478 Da were expressed at higher levels in the HC than in the CRC samples.

**Figure 1:**
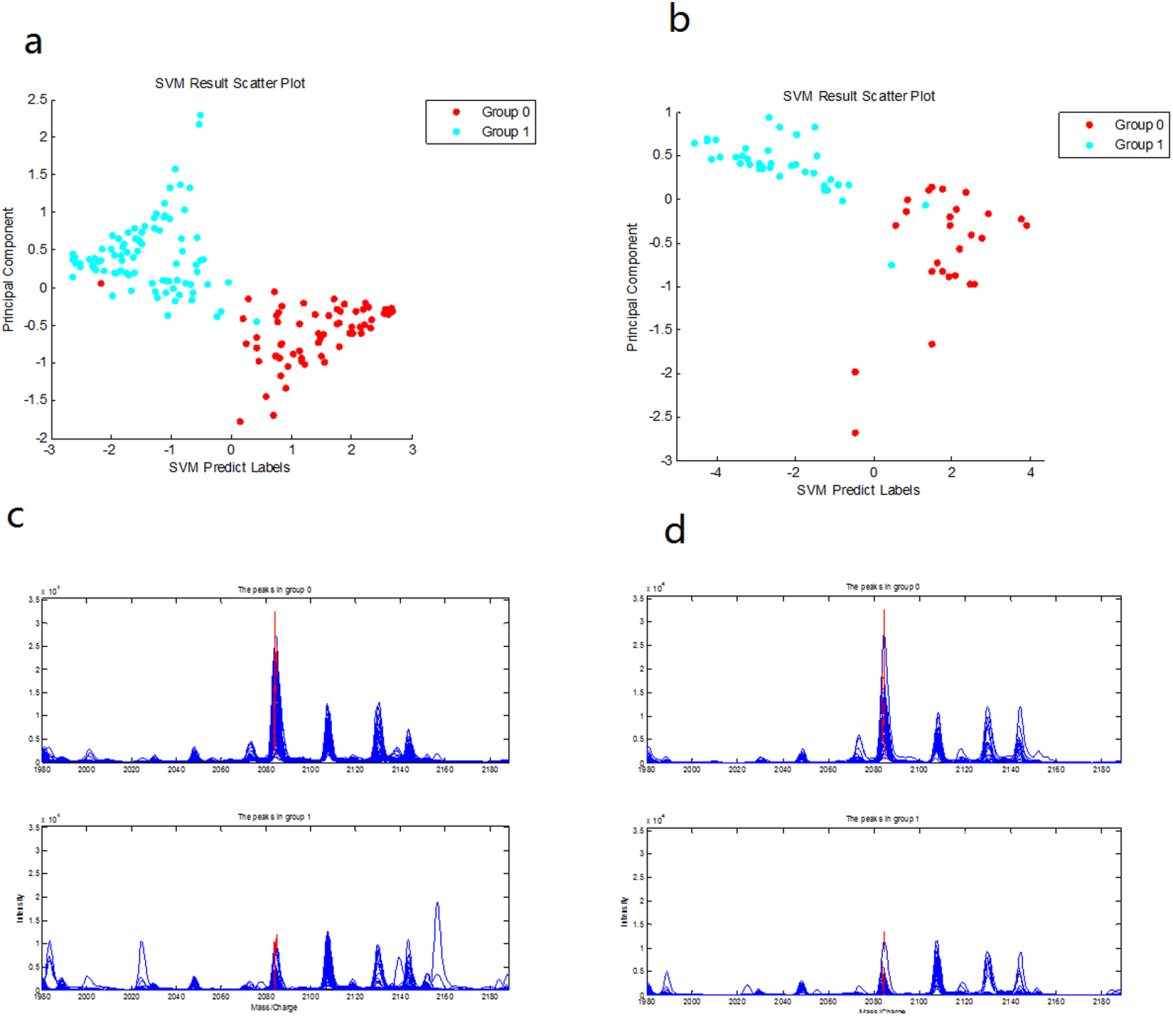
Biomarker SVM patterns. (**a**) Scatter diagram of the cross-validation results from the training set. (**b**) Scatter diagram of the results from the blind test set (Group 0 and 1 represent the HC subjects and CRC patients). (**c**) MST1 (2084 Da) expressed at low levels in the CRC group (group 1) in the training sets by MALDI-TOF-MS. (**d**) MST1 (2084 Da) expressed at low levels in the group CRC (group 1) in the test sets by MALDI-TOF-MS.

These peaks from the training set samples were selected, randomly combined, and input into the SVM pattern. The accuracy of all patterns was calculated, and the diagnostic pattern that achieved the highest Youden’s index was selected as the final pattern to distinguish CRC and HC samples. The pattern comprised 7 potential biomarkers with an *m/z* of 1781 Da, 1868 Da, 2084 Da, 1694 Da, 2886 Da, 2073 Da, and 4478 Da (Table 1). The SVM pattern composed of 7 peaks had a specificity of 99% and a sensitivity of 98%, as evaluated by a leave-one-out cross-validation. The remaining 65 serum samples, as a blind test set, were analysed on the second day to test the above SVM pattern. The sensitivity and specificity of the pattern blind test were 93% and 95%, respectively. (Table 2 and Fig. 1a,b).

**Table 1:** Protein peaks selected for the pattern to compare CRC patients and HC samples.

Biomarker (m/z)	*p*-value	Mean in HC for training	Mean in CRC for training	Mean in HC for testing	Mean in CRC for testing
1781	9.45E-23	219.53	2314.12	141.80	2249.97
1868	1.37E-21	374.89	3534.34	208.64	2972.30
2084	4.09E-20	9603.80	1845.85	9406.46	1836.56
1694	4.95E-18	39.83	706.14	22.86	692.00
2886	1.24E-15	2775.09	1671.69	2544.17	1774.45
2073	4.57E-14	916.50	259.62	1104.23	244.11
4478	1.27E-13	4152.99	1557.13	3824.15	1390.04

**Table 2:** Prediction results from the training and blind test cases using the established diagnosis pattern.

Groups		Total samples	Prediction Results		Accuracy (%)	
			Correct cases	Misjudged cases	Sensitivity (%)	Specificity (%)
Training	HC	63	62	1	98.4	/
	CRC	89	88	1	/	98.9
Blind Test	HC	26	24	2	92.3	/
	CRC	38	36	2	/	94.7

### Identification of candidate biomarkers

**Figure 2:**
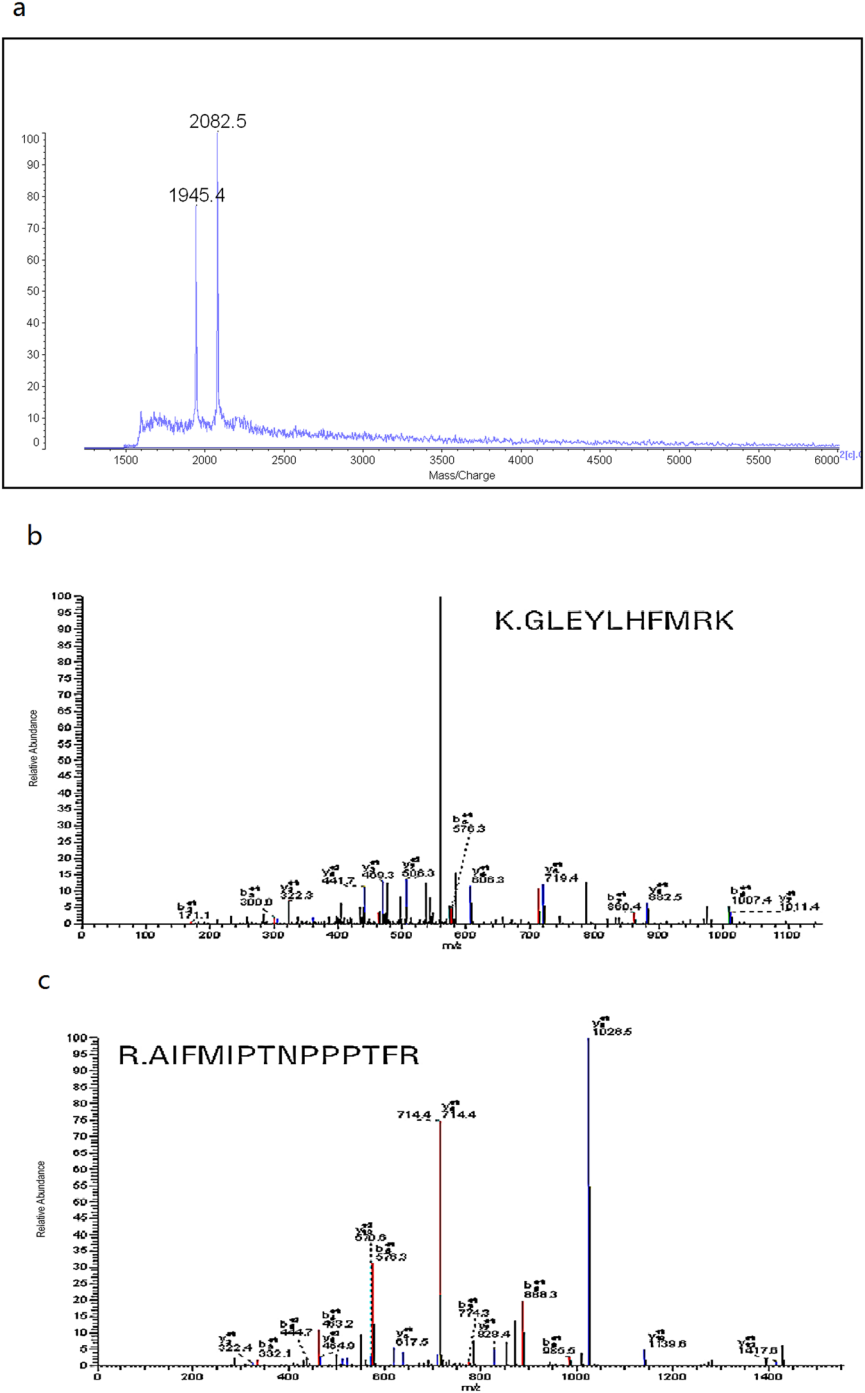
Purification and identification of MST1 (2084 Da). (**a**) Purification and detection data of the 2084 Da target biomarkers by MALDI-TOF-MS. (**b**) MS/MS spectra of K.GLEYLHFMR.K in the target biomarkers. (**c**) MS/MS spectra of R.AIFMIPTNPPPTFR in the target biomarkers.

The 2084 Da biomarkers that were expressed in low levels in CRC (Fig. 1c,d) in the pattern were separated and purified by HPLC (Fig. 2a).

The peptides were digested using trypsin and identified by MS/MS. The MS/MS data were searched using the BioWorks 3.3.1 software (Thermo, USA). Two peptide fragments with high confidence were found as K.GLEYLHFMR and R.AIFMIPTNPPPTFR. These two peptide fragments were matched with serine/threonine kinase 4 (STK4, also known as MST1; Fig. 2b,c and Table 3). In our premiere studies using SELDI-TOF-MS, MST1 was also determined to be expressed at low levels in the sera of CRC patients with an MZ of 3940 Da^32^.

**Table 3:** Structure data of the target protein.

Peptides identified	*p*	sf
K.GLEYLHFMR	1.8E-004	0.81
R.AIFMIPTNPPPTFR	2.5E-005	0.85

The value of *p* indicates the probability of protein. The score of sf indicated the quality of match.

### Validation of MST1 expression by western blotting

**Figure 3:**
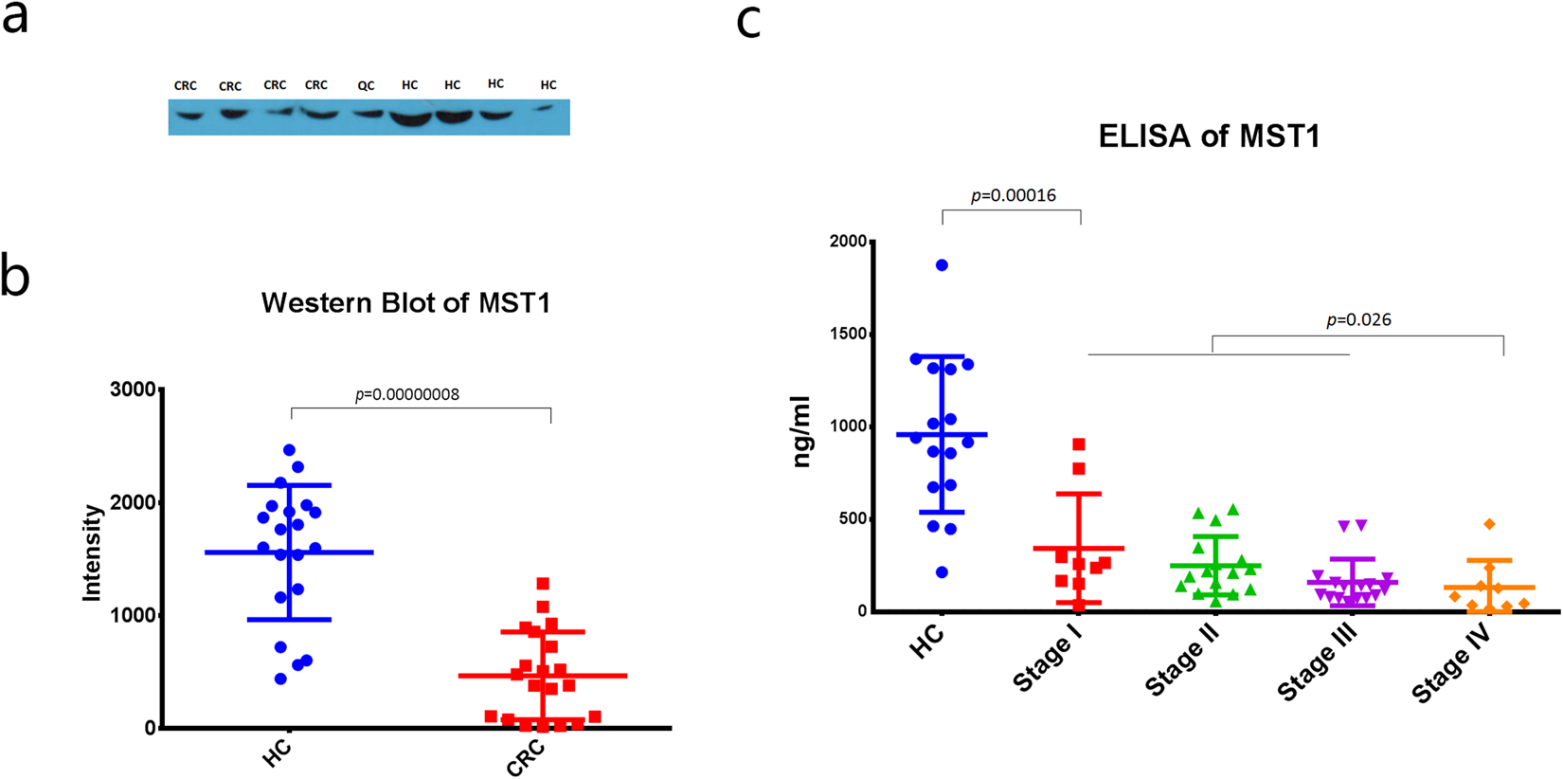
Verification results of the MST1 protein. (**a**) Representative western blotting image of MST1 in the CRC and HC sera in one PVDF membrane. Full-length blots are presented in Supplementary Figure S2. (**b**) Scatterplot of the normalized western blotting intensity of MST1 in 20 CRC patients and 20 HCs. (**c**) Scatterplot of the MST1 ELISA concentrations in the HC samples and the CRC samples at different stages.

To confirm the identity of the MST1 protein, an immunoassay was performed using western blotting and ELISA to detect the expression of MST1 in serum. Forty serum samples including 20 CRC patients and 20 healthy control individuals were selected for the western blotting. The gray value analysis intensity of MST1 expression in samples from different PVDF membranes was normalized to the same quality control (QC, pooled by all 40 samples). The normalized western blotting intensity of MST1 in the CRC patients (468.47 ± 390.35) were significantly lower (*p* = 7.79E-08) than in the healthy controls (1562.25 ± 594.96) (Fig. 3a,b). These results indicated that the expression levels of MST1 in the HC sera were higher than those in the CRC sera, which were in accord with the results from the MALDI-TOF-MS.

### Validation of MST1 expression by ELISA

Another 67 serum samples were selected, including 16 from healthy controls and 51 from colorectal cancer patients. These samples were analysed using an MST1 ELISA Kit to validate the intensity of MST1 in the serum samples. The CEA concentrations were also measured. The mean concentration of MST1 detected in the healthy controls (959.65 ± 407.07 ng/ml) was significantly higher (*p* = 1.87E-06) than that in the CRC patients (242.80 ± 261.99 ng/ml) and significantly higher (*p* = 0.00016) than that in the stage I CRC patients (343.97 ± 277.76 ng/ml), thus indicating its potential for use in the early diagnosis of CRC (Fig. 3c). In addition, the levels of MST1 were lower (*p* = 0.026) in stage IV CRC patients (132.49 ± 138.36 ng/ml), which exhibit distant metastasis, than the mean value in stage I, II and III CRC patients (266.44 ± 275.82 ng/ml) (Fig. 3c).

### Evaluation of the diagnostic value of MST1

According to the results, the expression levels of MST1 in the healthy control sera were higher than those in the colorectal cancer sera. Moreover, the concentration of MST1 decreased as the CRC patients advanced from stage I to stage IV. This result may indicate that MST1 is not only a biomarker for CRC detection but also a biomarker associated with the malignancy grade of CRC.

**Figure 4:**
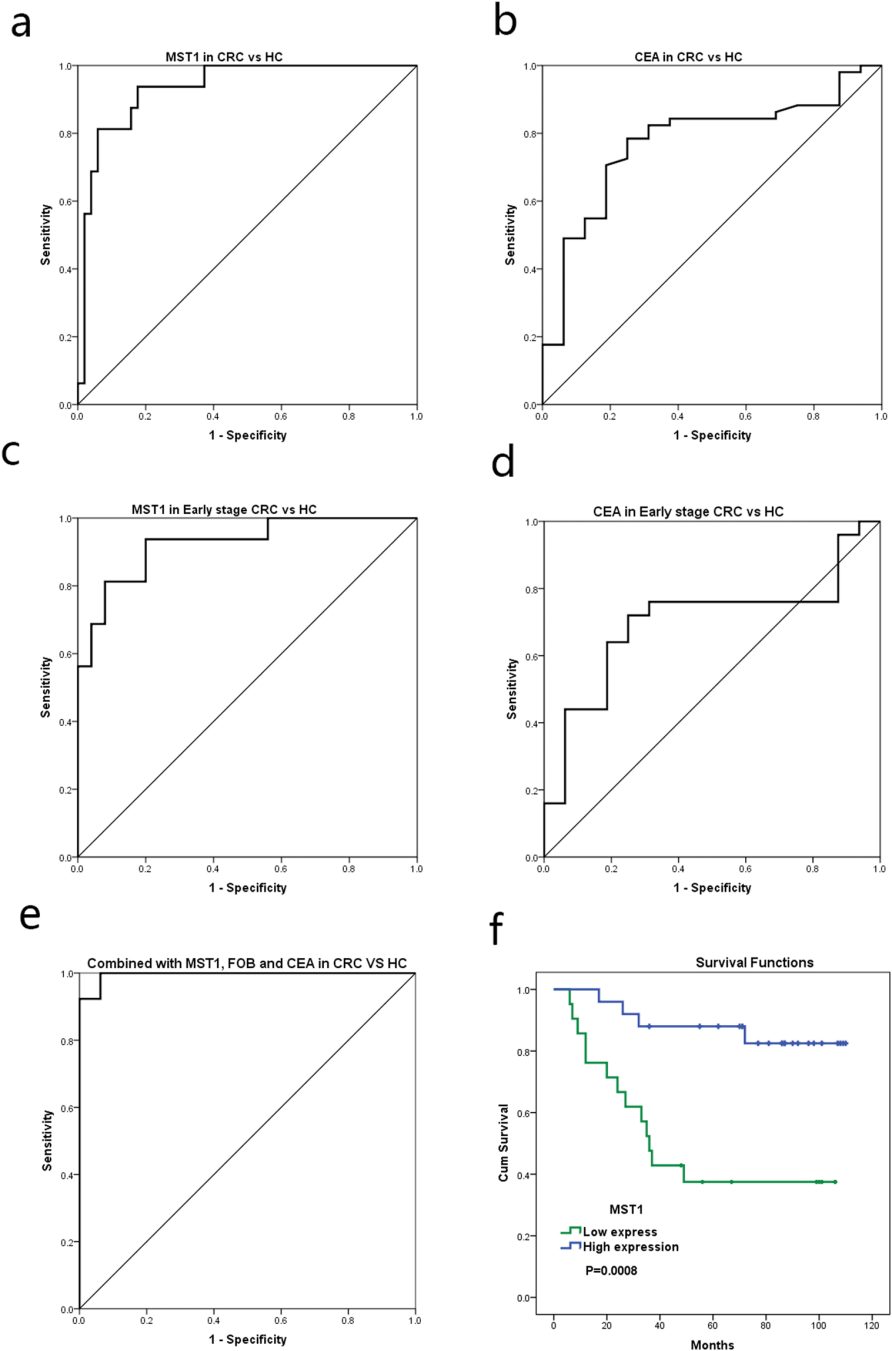
ROC curve of MST1 and CEA. (**a**) ROC curve for serum MST1 in CRC and HC. (**b**) ROC curve for serum CEA in CRC and HC. (**c**) ROC curve for serum MST1 in early stage CRC (stage I and II) and HC. (**d**) ROC curve for serum CEA in early stage CRC (stage I and II) and HC. (**e**) ROC curve for MST1 Combined with CEA and FOBT. (**f**) Kaplan–Meier survival analysis for MST1.

To evaluate the diagnostic value of MST1, a receiver operating characteristic (ROC) curve analysis was performed. The AUC for serum MST1 associated with a diagnosis of CRC was 0.934 (Fig. 4a). On the basis of the ROC curves, a serum MST1 concentration of 398.50 µg/ml was selected as the optimal cutoff value for differentiating CRC patients and controls, with a sensitivity of 82.4% and specificity of 93.8%, respectively.

The preoperative CEA value and FOBT were also detected in these samples. The AUC of CEA was 0.773 in the ROC curve analysis (Fig. 4b). The sensitivity was 37.3%, and the specificity was 93.8% with a cutoff value of 5 µg/l. And for FOBT, the sensitivity and specificity was 64.1% and 100%.

The diagnosis of MST1 Combined with CEA and FOBT in colorectal cancer was analysed by logistic regression and ROC curve. The sensitivity and specificity of the combination were 92.3% and 100%, respectively. The AUC of Combination is 0.995 (Fig. 4e). To evaluate the early diagnosis of MST1 in CRC, serum samples from 25 early stage CRC (I, II stage) patients were compared with HC samples. The AUC of MST1 was 0.925, and the AUC of CEA was 0.7 for early stage CRC detection (Fig. 4c,d). The sensitivity for MST1 serum levels for CRC early detection was 80.0% and specificity was 93.8%; in contrast, the sensitivity of CEA was 32.0% and specificity was 93.8% (Table 4).

**Table 4:** Performance of MST1 and CEA in the diagnosis of CRC.

	AUC (95% CI)	Sensitivity (%)	Specificity (%)
MST1 in CRC vs HC	0.934 (0.871–0.997)	82.4	93.8
CEA in CRC vs HC	0.773 (0.647–0.899)	37.3	93.8
MST1 in early stage CRC vs HC	0.925 (0.842–1.000)	80.0	93.8
CEA in early stage CRC vs HC	0.700 (0.534–0.866)	32.0	93.8

CI, coefficient interval.

### MST1 expression in samples of prognosis and treatment of CRC

Gene expression was analyzed using Affymetrix U133plus2.0 microarrays on mRNA samples from an additional cohort of 46 fresh CRC tissues before surgery, including 3 of stage I, 15 of stage II, 25 of stage III and 3 of stage IV, with a median follow-up period of 70.5 months^14^. Patient follow-up was conducted by a combination of phone calls and letters. Overall survival was defined as the time from completion of surgery to final follow-up date or death of the patient. MST1 mRNA expression level was evaluated using 7 microarray probe sets annotated to STK4. ROC curves were established for determining cutoff values of high MST1 expression group (n = 21) and low MST1 expression group (n = 25) for prediction of CRC prognosis (alive or dead) using Youden’s index. The Kaplan–Meier method with log-rank test was used to determine the cumulative probability of overall survival. Kaplan–Meier survival analysis indicated that low MST1 expression was associated with shortened survival (*p* = 0.0008; Fig. 4f). Furthermore, among the 46 CRC patients, 16 cancer deaths occurred within 5 years, and 25 patients lived for over five years after diagnosis. The group of cancer death within 5 years showed significantly lower MST1 expression than the group lived over five years after diagnosis (*p* = 0.0027).

The sera of 23 CRC patients before treatment (before chemotherapy or before surgery) and after treatment (one month after chemotherapy or surgery) were measured by ELISA (Table S1). The mean MST1 values In 23 CRC patients before and after treatment were 290.97 ± 164.25 and 365.15 ± 301.99, respectively. There was no significant difference in the level of MST1 in CRC patients before and after treatment (*p* = 0.098; Fig. S1). However, the values of MST1 were elevated in 65.22% (15/23) of the CRC samples after treatment.

Thirty-six other tumor patients’ sera (12 lung cancers, 12 gastric cancers, 12 esophageal cancers) were measured by ELISA to have the MST1 value of 246.20 ± 70.05, which have no difference comparing to colorectal cancer patients (*p* = 0.11; Table S1; Fig. S1).

## Discussion

Early diagnosis is crucial for improving the survival rate and the treatment effects of CRC patients. Several cancer biomarkers such as CEA and CA19-9 are used for the diagnosis, screening and treatment of CRC because of their accessibility and the minimal pain caused by serum assays. However, these tumor markers are relatively limited due to inadequate sensitivity or specificity. Discovering new biomarkers for diagnosis of CRC is still necessary.

Proteomic approaches are widely used in the discovery of cancer biomarkers. Two-dimensional polyacrylamide gel electrophoresis (2D-PAGE) can identify protein expression in serum, saliva, or tissue specimens^33^. MALDI-TOF-MS technology in proteomic has the advantages of being rapid, having a high sensitivity, and allowing for high-throughput analysis^34,35^.

In our Zhejiang University Proteomics Data Analysis System (ZJUPDAS) analysis, denoising with the Undecimated Discrete Wavelet Transform (UDWT) is likely to identify most of the true, reproducible peaks. In addition, SVM can resolve problems such as the generalization of the medium and small samples in the pattern recognition and pattern selection and over-fitting. Genetic algorithm (GA) can find the local optimal solution for the combination of multiple peaks. In addition, a leave-one-out cross- validation and independent test sets were used to obtain a robust proposed diagnostical model. These steps in the ZJUPDAS analysis ensure that the selection of biomarkers is not influenced by systematic bias^36,37^.

In our study, MALDI-TOF-MS and magnetic beads were used to analyse 217 serum samples, including 127 CRC and 90 HC samples. The diagnosis pattern consisting of seven biomarkers was able to discriminate CRC from HC samples with a specificity of 99% and sensitivity of 98%. In a blind test, the specificity reached 95%, and the sensitivity reached 93%. These results indicated that this diagnostic pattern could successfully diagnose CRC with high specificity and sensitivity. Furthermore, the 2084 Da peak in the diagnostic pattern was separated by HPLC and identified by liquid chromatography-Tandem mass spectrometry (LC-MS/MS) as the protein MST1. In our previous study, we have used SELDI-TOF-MS to identify a peptide that has low expression in colorectal cancer and a molecular weight of 3940 Da, which is also a part of the MST1 protein^32^. The other 6 peaks still require identification in our future studies.

Cleavage of Mst1 by caspase-3 can phosphorylate H2B at S14, and the phosphorylation is related to apoptotic chromatin^38^. MST1/2 is an important suppressor in the Hippo pathway^39^. Activated Mst1/2 phosphorylates Yes-associated protein (Yap) and decreases the abundance of Yap^40^. The Hippo signalling pathway is related to cell death, cell proliferation and tissue growth and is associated with various human cancers, including colorectal cancers^41,42^. Mst1/2 protein kinases restrain intestinal stem cell proliferation and colonic tumourigenesis by inhibiting the overabundance of Yap^43^. Mst1/2 may suppress tumour initiation^44^. Although up-regulation of MST1 has been observed in some cancers^45,46^, MST1 has been reported to be down-regulated in gastric cancer and exert a tumour suppressive function^47^. Our results also show MST1 is down-regulated in colorectal cancer of the gastrointestinal tract.

The cutoff value of 398.50 µg/ml for MST1 in serum differentiated CRC patients and controls with 82.4% sensitivity and 93.8% specificity. The accuracy was significantly better than that for CEA. And the sensitivity and specificity of MST1 combined with CEA and FOBT in diagnosis of colorectal cancer were 92.3% and 100%, respectively.

In summary, a diagnostic pattern was established and was able to distinguish CRC patients from HC subjects with high specificity and sensitivity. MST1 was identified and indicated to be an early detection protein biomarker, even for stage I CRC, and a predictive marker for distant metastasis of CRC. MST1 expression was down-regulated in CRC patients compared with HC subjects. Additionally, low MST1 mRNA expression was associated with poor prognosis. These results illustrated that MST1 might be an early detection protein biomarker for CRC and a predictive marker for distant metastasis and prognosis of CRC. However, our results also indicated that down-regulated expression of MST1 in many cancers, including lung cancer, gastric cancer, esophageal cancer and CRC. Therefore, MST1 may be not only a biomarker for CRC. In order to determine potential of our findings for clinical applications in colorectal cancer, further study with a larger sample size is needed.

## Materials and Methods

### Patients

This study was approved by the Institutional Review Board of Medical Research of The Second Affiliated Hospital, Zhejiang University School of Medicine, and all experiments were carried out in accordance with the approved guidelines. A total of 324 serum samples were collected from CRC patients (n = 198) and healthy individuals (n = 126) who had provided informed consent, at the Second Affiliated Hospital, Zhejiang University School of Medicine (Table 5). Sera from CRC patients were collected before the patients were treated with a surgical operation and chemotherapy, and the CRC diagnosis was confirmed by pathological diagnosis. These CRC patients included 166 with an adenocarcinoma, 15 with a mucinous adenocarcinoma, 3 with a signet-ring cell carcinoma, and 6 with a papillary adenoma and 8 with a tubular adenoma. The clinical stage of the CRC patients was 38 stage I, 59 stage II, and 71 stage III/IV. The average age of the patients was 61.0 y, and the healthy controls were age- and sex- matched. Within these samples, 127 CRC serum samples and 90 healthy control serum samples were analysed with MALDI-TOF-MS to identify new biomarkers. The remaining 107 samples were selected to verify the biomarkers with western blotting and ELISA. The preoperative sera were collected in the morning with limosis. The Sera were separated and stored at frozen −80 °C.

**Table 5:** Serum samples information.

Samples	MALDI-TOF-MS	Western blotting	ELISA
**Healthy Control**			
Total Number	90	20	16
Male/Female	48/42	11/9	7/9
Average age, y	61.3	59.6	62.4
**Colorectal Cancer**			
Total Number	127	20	51
Male/Female	70/57	10/10	28/23
Average age, y	60.5	62	61.7
Stage (I/II/III/IV)	25/36/48/18	3/7/8/2	10/16/15/10

### Magnetic beads-based sample preparation for MALDI-TOF-MS

We used MALDI-TOF-MS and magnetic beads to screen the serum samples to identify new biomarkers for colorectal cancer. The Sera were centrifuged at 10,000 g for 3 min at 4 °C after thawed on ice. Weak cation-exchange (WCX) magnetic beads (Bruker Daltonics, USA) were selected for the protein separation. According to the manufacturer’s protocol, first, 5 μL of serum was added to the mixture of 10 μL of beads slurry and 10 μL of binding solution in a 0.2-mL polypropylene tube. After incubation for 5 min, the supernatant was discarded. Then, the magnetic beads were washed with 100 μl wash buffer three times. Finally, the proteins were eluted from the magnetic beads with 5 μL elution buffer and mixed with 5 μL stable buffer. Finally, 1 μL protein mixture was deposited on a Micro SCOUT Plate (MSP). After natural drying, 1 μL CHCA matrix (3 mg/ml cyano-4-hydroxycinnamic acid, 50% ACN, 20% TFA) was added to each spot for detection.

### Data processing and analysis

Spectra were acquired for all samples in parallel using MALDI-TOF-MS (Microflex; Bruker Daltonics, USA) with the following settings: ion source 1, 20 kV; ion source 2, 18.4 kV; lens voltage 7.5 kV; and pulsed ion extraction, 120 ns. Irradiation with a nitrogen laser operated at 25 Hz and 30% laser power was used to achieve ionization-. Mass spectra were detected in linear positive mode, and protein peaks in the mass range 900–10,500 Da were recorded with FlexAnalysis acquisition software (Bruker Daltonics, USA). The initial data were exported as mzXML files using CompassXport software (Bruker Daltonics, USA).

MzXML files were imported and analysed in the ZJUPDAS, which was designed by Yu JK. ZJUPDAS and detailed protocols have been described in our previous report^48^. Aligned spectra were digitally processed by wavelet denoising and baseline correction using the wavelet transform method. Wilcoxon rank-sum tests were used to compare the mean normalized intensities between the CRC and HC samples. The biomarkers with *p* < 0.01 were selected. These markers were combined randomly to establish patterns by using GA and evaluated with SVM. The SVM classifier with a radial based function kernel was used to discriminate among the different groups. A leave-one-out cross-validation approach was applied to estimate the accuracy of the classifier. The SVM pattern with the local optimal accuracy solution was selected to be the final pattern, and the markers in this SVM pattern were selected as the set of potential biomarkers.

### Purification and Identification of candidate protein biomarker

The candidate protein in serum was purified using HPLC. The fraction with the candidate protein was collected after analysed by MALDI-TOF-MS, and digested with Trypsin. The protein was identified by MS/MS. The detailed methods of purification and identification were described in our previous study^32^.

### Verification of biomarker using western blotting and ELISA

The serum samples from 20 CRC patients and 20 HC samples were collected and subjected to western blotting analysis (Details in Table 1). In each well, 1 μl of serum was loaded and was run in 10% tris-glycine SDS buffer for 2 hours at 120 V. Four CRC samples, 4 healthy controls and a standard quality control were added onto one gel. The proteins were transferred to a PVDF membrane in transfer buffer at 250 mA for 2 hours. After blocking of the membrane with 0.5% skim milk in TBST for 1 hour, the membrane was incubated with the MST1 rabbit monoclonal antibody diluted 1:5000 at 4 °C overnight and then washed 3 times for 15 min each in TBST. The second HRP-coupled anti-rabbit antibody diluted 1:10,000 was added to the membrane and incubated for 1 hour at room temperature. After the membrane was washed 3 times for 15 min each in TBST, the detection of the proteins was carried out by application of ECL solution to the membrane and artificial exposure.

Another 67 serum samples including 16 healthy control and 51 colorectal cancer samples (10 from stage I, 16 from stage II, 15 from stage III, 10 from stage IV) were analysed using an MST1 ELISA Kit (Abcam, England) according to the manufacturer’s instructions. Standard MST1 dilutions (0, 8.32, 24.69, 74.07, 222.2, 666.7 and 2,000 pg/ml) were prepared, and triplicate 100 μl samples at each concentration were added to a 96-well plate. The serum was diluted 1:400 with solution A from the MST1 ELISA Kit, and triplicate 100-μl serum samples were added to the same plate. After incubation at room temperature for 2.5 hours, the supernatant in each well was removed. After the plates were washed four times, the biotinylated MST1 detection antibody (100 μl) was added to each well and incubated at room temperature for 45 min with gentle shaking. After washing, the TMB One-Step Substrate Reagent was added for visualization, and the samples were incubated at room temperature for 30 min. After addition of the stop solution, the optical density was immediately measured at 450 nm, and a standard curve was generated. The concentration of each sample was calculated according to the standard curve.

### Statistical analysis

SPSS Statistics 20.0 (IBM, Armonk, NY, USA) was used to perform the statistical analysis. Statistical tests were two sided, and *p* < 0.05 was considered statistically significant. Chi-square tests were applied to compare categorical variables. Two-tailed Student’s t-tests and one-way ANOVA were used to compare quantitative data.

## Electronic supplementary material

Supplementary Information
