# Supplementary material for: Identification of MST1 as a potential early detection biomarker for colorectal cancer through a proteomic approach
Source: Sci Rep. 2017 Oct 27;7:14265. doi: 10.1038/s41598-017-14539-x (PMC5660227; doi:10.1038/s41598-017-14539-x)
Supplement: Supplementary file 1 — Supplementary Information [file 41598_2017_14539_MOESM1_ESM.pdf]

Identification of MST1 as a potential early detection biomarker for colorectal cancer through a proteomic approach

Jiekai Yu[1 +], Xiaohui Zhai[2,1+], Xiaofen Li[1],Chenhan Zhong[1], Cheng Guo[1], Fuquan Yang[4], Ying Yuan[3], Shu Zheng[1,5]*

[1] Cancer Institute (Key Laboratory of Cancer Prevention and Intervention, China National Ministry of Education), The Second Affiliated Hospital, School of Medicine, Zhejiang University, Hangzhou, 310009, China

[2] Department of Medical Oncology, The Sixth Affiliated Hospital of Sun-Yat Sen University, Guangzhou, 510655, China

[3] Department of Medical Oncology, The Second Affiliated Hospital, School of Medicine, Zhejiang University, Hangzhou, 310009, China

[4] Proteomic Platform, Institute of Biophysics, Chinese Academy of Sciences, Beijing, 100101, China

[5] Research Center for Air Pollution and Health, School of Medicine, Zhejiang University, Hangzhou, 310058, China

[+] These authors contributed equally to this work.

Corresponding address: Professor Shu Zheng, Cancer Institute, the Second Affiliated Hospital, Zhejiang University School of Medicine, China. Tel.: +086-571-87784501, E-mail: zhengshu@zju.edu.cn

Supplementary Information

Supplementary Figure S1: Scatterplot of the MST1 ELISA concentrations in sera samples of lung cancer, gastric cancer, esophageal cancer, CRC before treatment and CRC after treatment.

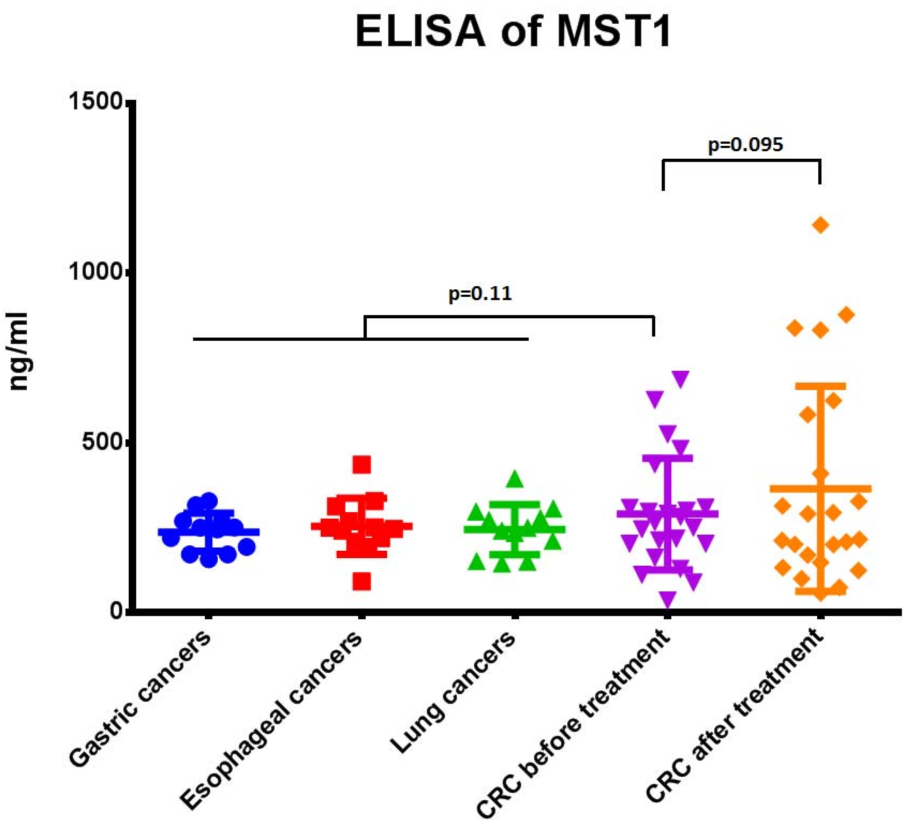

Supplementary Figure S2: Full-length blots of representative western blot image.

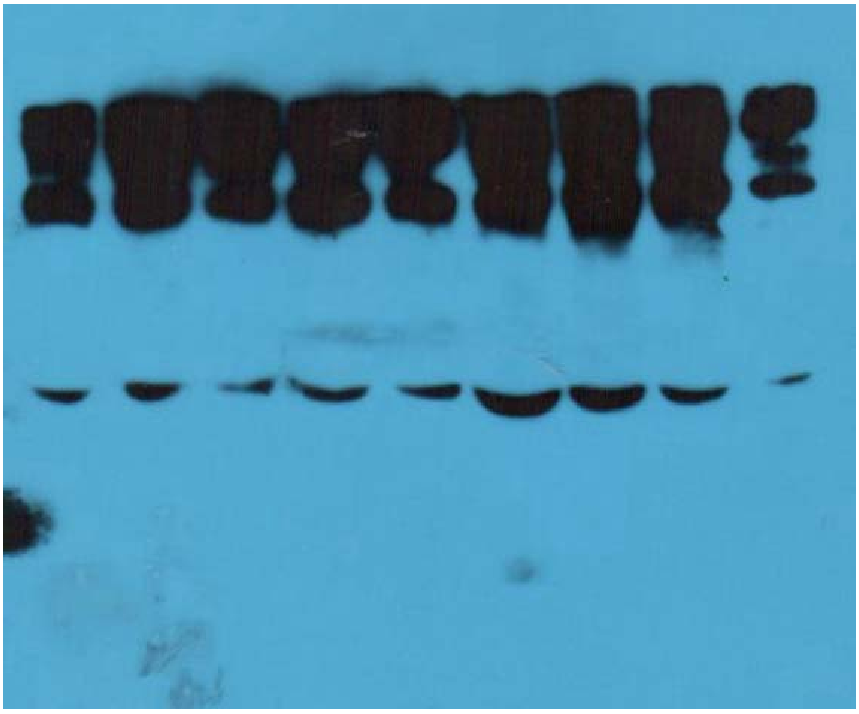

Supplementary Table S1. Information of CRC samples and other cancers samples.

Samples	Colorectal Cancer	Stomach cancer	Esophageal cancer	Lung cancer
Male / Female	13/10	3/9	9/3	3/9
Average age,y	54.10	59.92	59.08	63.75
Stage (I/II/III/IV)	1/3/8/11	2/4/4/2	2/4/4/2	3/1/5/3
Total Number	23	12	12	12
